# The VA MISSION Act of 2018: A Potential Game Changer for Rural GME Expansion and Veteran Health Care

**DOI:** 10.1111/jrh.12360

**Published:** 2019-03-08

**Authors:** Anthony P. Albanese, Edward T. Bope, Karen M. Sanders, Marjorie Bowman

**Affiliations:** ^1^ Office of Academic Affiliations US Department of Veterans Affairs Washington District of Columbia; ^2^ Northern California Healthcare System US Department of Veterans Affairs Sacramento California; ^3^ Department of Internal Medicine, Division of Gastroenterology/Hepatology UC Davis School of Medicine Sacramento California; ^4^ Department of Family Medicine The Ohio State University Columbus Ohio; ^5^ Department of Family Medicine Ohio University College of Osteopathic Medicine Athens Ohio; ^6^ Department of Internal Medicine, Division of Rheumatology Virginia Commonwealth University School of Medicine Richmond Virginia; ^7^ Department of Family Medicine University of Pennsylvania Philadelphia Pennsylvania

**Keywords:** graduate medical education (GME), health disparities, physician supply, policy, veterans

The Veterans Healthcare Administration (VHA) is the largest health care delivery system in the United States. It includes 172 medical centers (VAMCs), with over 1,100 Community Based Outpatient Centers (CBOCs) and various other sites of care. In addition to training physicians to care for veterans and active duty service members, the VHA is also directed to assist in supplying health care personnel to the nation.

Through its Office of Academic Affiliations (OAA), VHA develops partnerships with Accreditation Council for Graduate Medical Education (ACGME)/American Osteopathic Association (AOA) accredited residency program sponsoring institutions. These collaborations include 144 out of 152 allopathic medical schools and all 34 osteopathic medical schools. Through partnerships, in 2018, VHA provided training to 45,296 medical residents, 24,643 medical students, and many other associated health profession trainees.^1^

The gap between supply and demand of physicians continues to grow.^2^, ^3^ This is particularly significant in rural areas. US Census data show that a higher percentage of veterans live in rural areas than all Americans (24% vs 19.3%).^4^, ^5^ Compared to urban veterans, they experience higher disease prevalence and lower physical and mental quality‐of‐life scores.^6^ Addressing the problem of physician shortages is a mission‐critical priority for the VHA.^7^ In response, Section 301b of the Veterans Access, Choice, and Accountability Act of 2014 (VACAA),^8^ amended by Section 617 of the Veterans Healthcare Benefits Improvement Act of 2016,^9^ requires VHA to fund up to 1,500 new graduate medical education (GME) positions by August 7, 2024. Although VHA has already approved 1,055 of these, only 67.5 resident positions (6.4%) are in self‐designated rural sites. The VACAA effort has revealed difficulty in expanding GME in rural areas due to a dearth of partnership options, infrastructure funding difficulties, and a lack of incentives for residents to train in rural and underserved areas.^10^

In June of 2018, Public Law 115–182, known as the John S. McCain III, Daniel K. Akaka, and Samuel R. Johnson VA Maintaining Internal Systems and Strengthening Integrated Outside Networks Act of 2018 (VA MISSION Act),^11^ was passed. The primary purpose of the VA MISSION Act is to establish an effective and more efficient community care program for veterans and create a framework through which to modernize and realign the resources of the VHA. The Act provides $5.2 billion to expand access to community care through the Veterans Choice Fund, while sunsetting some limitations described in section 101 of the VACAA (PL 113–146).^8^

The VA MISSION Act of 2018 has 2 distinct aspects. Two of the 5 titles aim to improve veteran access to care by expanding community options and creating a commission to modernize and streamline VHA infrastructure. The remaining 3 titles mandate innovations designed to redistribute medical personnel, specialized services, and GME to rural and underserved locations throughout the United States.

Title I focuses on improvements to health care delivery including expansion of telehealth authority, expansion of the family caregiver program, and a new authority to support the cost of live donor transplant care for veteran recipients. Provisions to improve communications, payments, and collections between VHA, insurers, and community providers are included, as are training, competency, and continuing education requirements for non‐VA health care professionals. It also authorizes creation of a “Center for Innovation for Care and Payment” to innovate through the use of pilot programs that could be extended or modified to improve quality, access, or cost savings.

Title II of the Act also establishes a commission to comprehensively review VHA infrastructure and assets, and to modernize, realign, and close facilities strategically. There has been some controversy because of concerns that VA facility closures could lead to loss of services for veterans in affected areas. Nevertheless, VA's large footprint of both buildings and land would likely benefit from a systematic review.

Title III offers new options for VHA to recruit physicians and dentists into rural and underserved areas. Two new scholarship opportunities and a Specialty Education Loan Repayment program are created, and funding is increased for the current Education Debt Reduction Program.

1) Health Professional Scholarship Program for Physicians and Dentists (HPSP). After regulations are established, 2‐ to 4‐year medical or dental school scholarships (tuition, fees, and stipend) will be offered in exchange for VHA service. This scholarship will function similarly to the HPSP offered through the Department of Defense (DoD), but with a longer repayment period (18 months/year for VHA vs 12 months/year for DoD). After completing the residency (and possibly fellowship) of their choice, participants would be given a VHA assignment in an area of the United States experiencing a critical need that matches their specialty training. The number of scholarships available will be based on VHA‐determined provider shortages. This authority is set to expire in 2035.

2) “Veterans Healing Veterans” Medical Access and Scholarship Program. This pilot program provides 4 years of tuition, fees, and stipend support for 2 veterans at each of the 5 Teague‐Cranston medical schools,^1^ and the 4 historically black medical schools^2^ in exchange for 4 years of clinical practice at a VA facility after completion of a residency and/or fellowship. The Teague‐Cranston Act of 1972 created 5 medical schools in conjunction with established VA Medical Centers to provide care to veterans and community members in rural and other medically underserved areas of the United States.

3) The Specialty Loan Repayment program offers $40,000 per 12 months of clinical service in VHA to physicians in residency training, with a maximum repayment of $160,000. To be eligible, residents/fellows must have 2 or more years remaining in training. Preference will be given to residents who are veterans, and residents training in rural areas, operated by Indian tribes, Indian Health Service, or affiliated with underserved health care facilities. The recipients could then select their location of service from a list of approved VHA facilities.

Title IV of the VA MISSION Act requires the development of criteria to designate VA Medical Centers as underserved facilities and a plan to address their needs. It also enables a pilot program to furnish mobile deployment teams of needed medical personnel to these facilities. In addition, Title IV, section 403 creates a pilot program with 2 new authorities to increase physician training in underserved areas.

1. The first authority enables VA‐paid residents to provide care in “covered” federal facilities outside of the traditional VA campus. Using positions created through VACAA, at least 100 residents will train in facilities of the Indian Health Service, tribal health care organizations, or designated underserved VA areas, augmenting both the current health care workforce, and creating a new workforce pipeline. Federally Qualified Health Centers and DoD medical centers are included on the list of covered facilities as potential resident training locations.
2. The second new authority gives VHA the ability to assist with development costs of new residency programs starting in VA‐designated underserved communities. It will take about 18 months to develop regulations that will guide the enactment of this portion of the legislation and determine how to evaluate these pilot projects.

Title V includes a potpourri of various non‐GME‐related topics including incorporating peer specialists within Patient Aligned Care Teams (PACT),^12^ clarifying the role of VA podiatrists, and a pilot project to use medical scribes to improve patient access in various medical settings. This section also modifies the definition of major facility projects, and it appropriates the aforementioned $5.2 billion to the Veterans Choice Fund.

Overall, the VA MISSION Act will be a “game changer” in bringing GME to rural areas. Titles III and IV specifically address difficulties identified during the implementation of the prior VACAA effort—namely, the complexity of expanding GME in rural areas due to a dearth of partnership options, infrastructure funding difficulties, and a lack of incentives for residents to train in rural and underserved areas.^10^ Given that physicians who choose to train in rural areas often remain in those areas for some time after completing their training,^13^ the VA MISSION Act should lead to a greater number of physicians to serve veterans in rural America. We (the authors) believe these new authorities can potentially improve rural continuity of care, aid VHA in filling chronically vacant physician positions, and create a new workforce pipeline to benefit both veterans and communities alike. The pilot projects in section 403 will allow VHA to explore funding GME training off the traditional VA campus at Indian Health Service, other tribal health care facilities, and other underserved veteran communities. Implementation of the programs described above is contingent on federal funding. The VA MISSION Act of 2018 brings welcomed flexibility to VHA in fulfilling its missions to “give veterans the highest quality medical care,” “affording the medical veteran the opportunity for postgraduate study, which he was compelled to forgo in serving his country,” and “raising generally the standard of medical practice in the United States by the expression of facilities for graduate education.”^14^

## Endnotes

1 Teague‐Cranston medical schools: 1) Texas A&M College of Medicine, 2) Quillen College of Medicine at East Tennessee State University, 3) Boonshoft School of Medicine at Wright State University, 4) Edwards School of Medicine at Marshall University, and 5) the University of South Carolina School of Medicine.

2 Historically black medical schools: 1) Drew University of Medicine and Science, 2) Howard University of Medicine, 3) Meharry Medical College, and 4) Morehouse School of Medicine.
